# Association between opioid use during mechanical ventilation in preterm infants and evidence of brain injury: a propensity score-matched cohort study

**DOI:** 10.1016/j.eclinm.2023.102296

**Published:** 2023-10-28

**Authors:** Lisa Szatkowski, Don Sharkey, Helen Budge, Shalini Ojha

**Affiliations:** a Centre for Perinatal Research, Lifespan and Population Health, School of Medicine, University of Nottingham, Nottingham, UK; b Neonatal Unit, University Hospitals of Derby and Burton NHS Trust, Derby, UK

**Keywords:** Infant, Preterm, Mechanical ventilation, Opioid, Neurodevelopment

## Abstract

**Background:**

Preterm infants often require mechanical ventilation (MV), which can be a painful experience. Opioids (such as morphine) are used to provide analgesia, despite conflicting evidence about their impact on the developing brain. We aimed to quantify the use of opioids during MV in infants born at <32 weeks' gestational age and to investigate the association between opioid use and evidence of brain injury.

**Methods:**

In this retrospective propensity score-matched cohort study, we used routinely recorded data from the National Neonatal Research Database to study infants born at 22–31 weeks gestational age who were admitted to neonatal units in England and Wales (between Jan 1, 2012, and Dec 31, 2020) and who were mechanically ventilated on one or more days during their hospital stay. We used propensity score matching to identify pairs of infants (one who received opioids during MV and one who did not) with similar demographic and clinical characteristics. The pre-specified primary outcome was preterm brain injury assessed in all infants who received MV for more than two days and had evidence of preterm brain injury at or before discharge from neonatal care. Adjusted analyses accounted for differences in infants’ characteristics, including illness severity and painful/surgical conditions.

**Findings:**

Of 67,206 infants included, 45,193 (67%) were mechanically ventilated for one or more days and 26,201 (58% of 45,193) received an opioid whilst ventilated. Opioids were given for a median of 67% of ventilated days (IQR 43–92%) and the median exposure was 4 days (2–11). The percentage of mechanically ventilated infants who received opioids while ventilated increased from 52% in 2012 to 60% in 2020 (morphine, 51%–56%; fentanyl, 6%–18%). In the propensity score-matched cohort of 3608 pairs who were ventilated for >2 consecutive days, the odds of any preterm brain injury (adjusted odds ratio 1.22, 95% CI 1.10–1.35) were higher in those who received opioids compared with those who did not (received opioids, 990/3608 (27.4%) vs. did not receive opioids, 855/3608 (23.7%). The adjusted odds of these adverse outcomes increased with increasing number of days of opioid exposure.

**Interpretation:**

Use of opioids during mechanical ventilation of preterm infants increased during the study period (2012–2020). Although causation cannot be determined, among those ventilated for >2 consecutive days, these data suggest that opioid use is associated with an increased risk of preterm brain injury and the risk increases with longer durations of exposure.

**Funding:**

University of Nottingham Impact Fund.

**Research in context**

**Evidence before this study**

Mechanical ventilation is lifesaving, but uncomfortable and potentially painful, for preterm infants. Morphine and other opioids may provide some analgesia during ventilation. We performed a review of published English language literature up to August 2023 investigating the effect of opioid use on developmental outcomes in preterm infants. The impact of early life exposure to opioids on the developing brain is unclear. We aimed to investigate the association between use of opioids, duration of exposure, and evidence of preterm brain injury.

**Added value of this study**

Our findings, using data between 2012 and 2020, show that morphine and fentanyl use among mechanically ventilated preterm infants is increasing. Additionally, they suggest that morphine use in preterm infants during mechanical ventilation is associated with an increased risk of brain injury. Subanalyses showed that longer duration of exposure to opioids may increase this risk.

**Implications of all the available evidence**

Morphine and other opioids should be used judiciously in mechanically ventilated preterm infants, as exposure is associated with increased risk of brain injury irrespective of condition at birth and illness severity. Duration of use should be minimised as prolonged exposure may worsen this risk. Efficacy and safety of new drugs that could provide analgesia and reduce need for opioids should be investigated.

## Introduction

Over half of infants born at ≤32 weeks' gestational age (GA) are mechanically ventilated on the day of birth and many more require ventilatory support later during neonatal care.^1^ Mechanical ventilation (MV), and often the underlying condition that necessitated respiratory support, such as necrotising enterocolitis (NEC), can be painful. Although optimal management of pain during MV is as yet unclear, selective use of continuous infusion of opioids is recommended for infants on prolonged MV.^2^^,^^3^ Intravenous morphine or fentanyl is usually the drug of choice.^4^^,^^5^

The Cochrane review of opioids for infants receiving MV concluded that there is uncertainty whether opioids reduce pain in mechanically ventilated infants.^6^ Additionally, there may be short and long-term adverse consequences of morphine infusion in preterm infants, such as respiratory depression leading to prolonged duration of MV, slowing of gastrointestinal transit causing delays in establishing enteral feeds,^7^ and uncertain long-term neurodevelopmental effects.^8^ Post-hoc secondary analyses of data from the Preterm Erythropoietin Neuroprotection Trial showed that propensity score-matched extremely preterm infants who received opioids and/or benzodiazepines had worse neurodevelopmental outcomes at 2-years when compared with those who did not receive these drugs.^9^ Longer term outcomes of randomised controlled trials of morphine analgesia in mechanically ventilated preterm infants are conflicting^10^^,^^11^ and effect on the brain may be modulated by the infant's underlying illness and dose and duration of morphine exposure.^8^

In England and Wales, the percentage of infants born at ≤32 weeks' GA who receive intravenous (IV) morphine at any point during their neonatal care increased from 32% in 2010 to 37% in 2017 while use of fentanyl increased from 2.8% to 7.8%.^12^ However, no study has evaluated more recent trends in use of opioids during MV in neonatal care in this population, nor outcomes associated with exposure. We aimed to quantify the change in use of MV and concomitant opioid use in infants born at <32 weeks' GA between 2012 and 2020 in England and Wales and to investigate the association between use of opioids, duration of exposure, and evidence of preterm brain injury.

## Methods

### Study design and ethical approval

We used data from the National Neonatal Research Database (NNRD), as previously described,^1^ for infants born at 22–31 weeks' GA admitted to neonatal units in England and Wales from 01 January 2012 to 31 December 2020. The dataset was created by the NDAU and the study was approved by Yorkshire & The Humber—Sheffield Research Ethics Committee (IRAS 259802). The study protocol is provided in the Supplementary Materials.

### Exclusions

Infants were excluded if they had missing data on sex, birthweight, final discharge destination or had missing records for one or more episodes of care (i.e., we excluded those babies who were cared for in more than one hospital and records of care were not available from one or more hospitals where care was provided). We also excluded infants with implausible birthweight for GA z-scores >4 standard deviations (SD) or <−4 SD or who were admitted >24 h after birth and those who had neonatal abstinence symptoms or syndrome and/or maternal opioid (Supplementary Table S1). Post-hoc, we excluded those who were born with a congenital anomaly deemed to be invariably fatal (Supplementary Table S2).

### Exposures

From daily records, we identified infants who were mechanically ventilated on one or more days during their stay and identified the duration, in days, of the longest continuous course of MV. From daily records of prescribed drugs, we identified use of opioids (including IV and oral morphine, fentanyl, and its derivatives). To further understand the use of analgesics and sedatives in ventilated infants we also identified use of benzodiazepines and muscle relaxants as they are often used with opioids in ventilated infants (see Supplementary Table S3 for included drugs).

### Outcomes

The pre-specified primary outcome, based on the National Neonatal Audit Programme definition,^13^ was preterm brain injury, defined as intraventricular haemorrhage (IVH) of any grade identified by imaging on or before day 28, cystic periventricular leukomalacia (PVL) or post haemorrhagic ventricular dilatation (PHVD) identified by any imaging during neonatal stay. We also pre-specified several secondary outcomes, including the most severe brain injuries, a composite outcome of brain injury or death, and other outcomes included in the Neonatal Core Outcome Set^14^ (Supplementary Table S4).

### Statistical analysis

All data management and analyses were performed using STATA, version 17 (StataCorp, College Station, Tx). After exclusions, we quantified the percentage of admissions where infants were mechanically ventilated for one or more days, on their day of birth, and for >2 consecutive days. We then quantified the percentage of admissions where an opioid (morphine and/or fentanyl) was prescribed, and the percentage of infants who were mechanically ventilated for >2 consecutive days who received an opioid whilst ventilated. Data are presented for all infants as well as for two pre-specified subgroups: those born at <28 weeks' GA, and those born at 28–31 weeks' GA. To explore variation by neonatal unit of care, we calculated the percentage of ventilated days where infants received an opioid individually for all Level 3 (Neonatal Intensive Care Units) and larger Level 2 (Local Neonatal Units) units (defined as those who delivered at least 15,000 patient care days per year—approximately the median workload across all Level 2 units).

We compared the characteristics of infants who were mechanically ventilated for >2 consecutive days who did, and did not, receive an opioid during MV. Characteristics compared included demographic characteristics, characteristics of pregnancy and delivery, NMR-2000 score,^15^ need for inotropes during the MV episode, and whether the infant had a major congenital anomaly requiring early surgical intervention, or a record of a painful procedure or diagnosis requiring urgent surgery in the first 2 days of MV (see Supplementary Table S5 for included conditions). We selected those who received at least 3 days of MV to exclude brief periods of MV for which opioid infusions are less likely to have been used or if used, would be given for short durations which would be unlikely to impact the outcomes of interest.

We used propensity score matching to identify pairs of infants (one who received opioids during MV and one who did not) with similar demographic and clinical characteristics, using the methods outlined by Imbens and Rubin.^16^ We used nearest neighbour matching, with a calliper width of 0.2 times the standard deviation of the logit of the propensity scores.^17^ Detailed description of the propensity score matching methods is given in the supplementary material, including details of the background variables used to derive the propensity scores, which spanned a range of infant, mother and unit-level characteristics (Supplementary Table S6) and assessment of the quality of the match (Supplementary Figure S1 and Supplementary Table S7). We included risk of in-hospital mortality at birth (NMR-2000 risk category) to match infants on their condition soon after birth and the need for inotropes during the episode of MV to match how unwell they may have been when they did or did not receive opioids. Post-hoc, we included the variable indicating whether an infant had a condition requiring urgent surgical intervention and/or a record of a painful procedure or diagnosis in the first 2 days of MV in the propensity score estimation procedure to match infants on potential indication for opioid use.

In the resulting matched cohort, we used univariable logistic regression, with no further adjustment, to explore the association between receiving opioids during MV and the primary and secondary outcomes. We used a Bonferroni adjustment to account for multiple hypothesis testing. In a sensitivity analysis, we used overlap weights^18^ derived from the propensity scores to weight the calculation of odds ratios for adverse outcomes in the full cohort, such that all infants contributed to the analysis with their contribution proportional to their overlap weight.

### Analysis by duration of opioid exposure

We used multivariable logistic regression to examine the association between the number of days of exposure to opioids whilst ventilated and odds of preterm brain injury, severe brain injury, and the composite outcome of brain injury and/or death. Infants with zero days of exposure were the reference group. We analysed exposure for each extra day of exposure for one to 5 days. Those with more than 5 days of exposure were grouped due to relatively small numbers. Given the number of exposure groups, and small numbers in some groups, we were not able to use propensity score matching to adjust for confounders.

The logistic regression models were adjusted for a range of variables to account for differences in characteristics of infants, including sex, GA, birthweight-for-age z-score <−2SD, NMR 2000 risk category, 5 min Apgar score, unit level of first admission, year of birth, number of days of care, inotropes received whilst ventilated, and painful/surgical procedure or condition (as listed in Supplementary Table S5). We used robust standard errors to account for clustering by neonatal unit.

### Role of the funding source

The funder of the study had no role in study design, data collection, data analyses, interpretation, writing of the report, or the decision to submit for publication.

## Results

Of 68,934 infants born at 22–31 weeks' GA admitted from 2012 to 2020, 67,206 were included (Supplementary Figure S2). 45,193 (67.2%) were ventilated, including 38,166 (56.8%) who were ventilated on the day of birth and 24,815 (36.9%) who were ventilated for >2 consecutive days (Table 1).

**Table 1:** Opioid use during mechanical ventilation in infants <32 weeks' GA in England and Wales (2012–2020).

	All infants n = 67,206	<28 weeks' GA n = 21,043	28–31 weeks' GA n = 46,163
**Mechanical ventilation**			
Received any MV, n (%)	45,193 (67.2)	20,222 (96.1)	24,971 (54.1)
MV on day of birth, n (%)	38,166 (56.8)	18,827 (89.5)	19,339 (41.9)
MV for >2 consecutive days at least once, n (%)	24,815 (36.9)	15,477 (73.5)	9338 (20.2)
Number of days of MV, median (IQR)	4 (2–12)	11 (4–27)	2 (1–4)
Length (days) of longest continuous course of MV, median (IQR)	3 (2–8)	7 (3–19)	2 (1–4)
**Use of opioids**			
Any opioids, n (%)	26,900 (40.0)	14,675 (69.7)	12,225 (26.5)
Any morphine, n (%)	25,780 (38.4)	14,418 (68.5)	11,362 (24.6)
IV morphine, n (%)	25,594 (38.1)	14,294 (67.9)	11,300 (24.5)
Oral morphine, n (%)	3199 (4.8)	2761 (13.1)	438 (0.9)
Fentanyl, n (%)	5050 (7.5)	2736 (13.0)	2314 (5.0)
Cumulative days of opioid exposure, median (IQR)	4 (2–12)	8 (3–21)	2 (1–5)
Cumulative days of morphine exposure, median (IQR)	5 (2–13)	8 (3–21)	3 (1–5)
Cumulative days of fentanyl exposure, median (IQR)	1 (1–2)	1 (1–2)	1 (1–1)
**Opioids while on MV**			
Received any opioid, n (%)	26,201 (58.0)	14,536 (71.9)	11,665 (46.7)
Received morphine, n (%)	25,274 (55.9)	14,292 (70.7)	10,982 (44.0)
Received fentanyl, n (%)	4723 (10.5)	2642 (13.1)	2081 (8.3)
% of MV days any opioid received, median (IQR)	66.7 (42.9–91.7)	63.6 (36.4–85.7)	71.4 (50.0–100.0)
% of MV days morphine received, median (IQR)	66.7 (42.9–90.9)	63.3 (36.6–85.7)	71.4 (50.0–100.0)
% of MV days fentanyl received, median (IQR)	13.6 (5.5–33.3)	6.9 (3.6–14.3)	33.3 (16.7–50.0)

GA, gestational age; IQR, inter-quartile range; IV, intravenous; MV, mechanical ventilation.

The annual percentage of infants who were ventilated at any time decreased from 68.2% (5187/7600) in 2012 to 64.8% (4234/6532) in 2020. This included a decrease in those who were ventilated on day one (from 59.4% to 51.9%) and those ventilated for >2 consecutive days (from 37.7% to 36.7%). These figures are given by GA sub-group and year of admission in Supplementary Figure S3.

Overall, IV morphine was given to 38.1% (25,594/67,206) of infants, fentanyl to 7.5% (5050/67,206), midazolam to 3.3% (2226/67,206), and muscle relaxants to 13.6% (9123/67,206) infants. We did not find any record of use of methadone and sufentanil while remifentanil was given to 177 infants. Use of opioids, midazolam, and muscle relaxants by GA sub-group are given in Supplementary Table S8.

### Use of opioids in mechanically ventilated infants

**Fig. 1:**
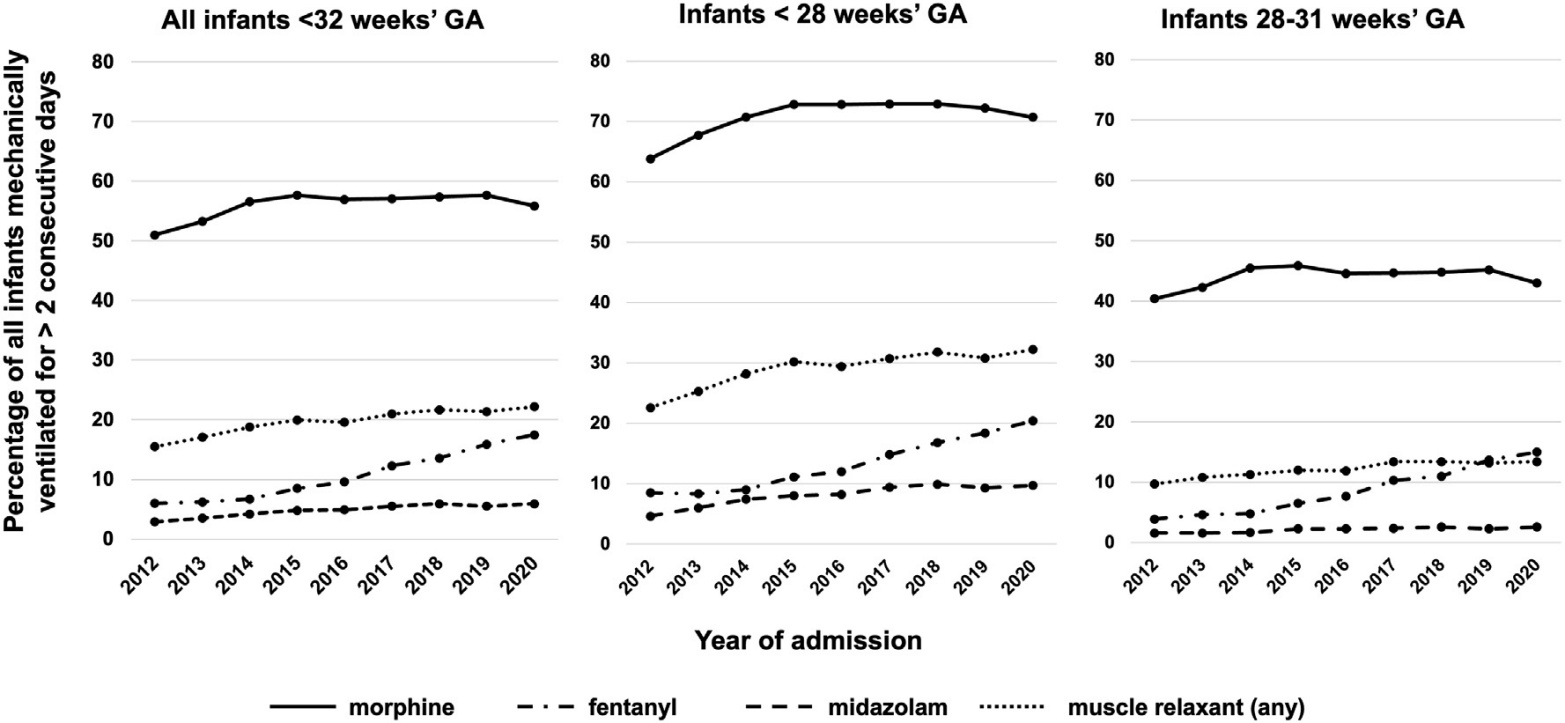
Trends in use of opioids, midazolam and muscle relaxants during mechanical ventilation in infants born at ≤32 weeks' gestational age in England and Wales (2012–2020), by gestational age sub-group.

Of the 45,193 infants who had MV, 55.9% (25,274) had morphine and 10.5% (4723) had fentanyl while on MV (Table 1). The percentage of mechanically ventilated infants who received these drugs during MV increased between 2012 and 2020 (morphine, 51.0%–55.9%; fentanyl, 6.0%–17.5%). Use of midazolam and muscle relaxants also increased over the study period (Fig. 1).

Baseline characteristics of mechanically ventilated infants who received opioids during MV and those who did not are given in Table 2.

**Table 2:** Characteristics of infants born at <32 weeks' GA and mechanically ventilated for >2 consecutive days who did and did not receive an opioid whilst ventilated.

	All infants	Received opioid	Did not receive opioid	p-value
Number of infants	24,815	20,561	4254	
Gestational age in weeks, median (IQR)	27 (25–28)	26 (25–28)	27 (26–29)	<0.0001
Birth weight in grams, median (IQR)	890 (710–1160)	880 (700–1150)	946 (760–1200)	<0.0001
Birth weight z-score, median (IQR)	−0.10 (−0.80 to 0.50)	−0.10 (−0.80 to 0.40)	−0.10 (−0.80 to 0.50)	0.27
Sex, n (%)				
Female	10,503 (42.3)	8620 (41.9)	1883 (44.3)	0.0049
Male	14,312 (57.7)	11,941 (58.1)	2371 (55.7)	
Multiple birth, n (%)	6100 (24.6)	4948 (24.1)	1152 (27.1)	0.00011
Any antenatal steroid, n (%)^a^	22,132 (89.7)	18,291 (89.4)	3841 (91.1)	0.00086
Caesarean delivery, n (%)^a^	12,906 (53.9)	10,573 (53.2)	2333 (57.3)	<0.0001
Early onset sepsis, n (%)	15,199 (61.3)	13,150 (64.0)	2049 (48.2)	<0.0001
Surfactant given on day 1, n (%)^a^	21,143 (88.2)	17,768 (88.7)	3375 (85.7)	<0.0001
Mechanical ventilation on day 1, n (%)	21,636 (87.2)	17,966 (87.4)	3670 (86.3)	0.49
Received inotropes whilst ventilated, n (%)	12,745 (51.4)	11,707 (56.9)	1038 (24.4)	<0.0001
Number of days mechanical ventilation, median (IQR)	10 (5–25)	12 (6–27)	6 (4–11)	<0.0001
Major congenital anomaly requiring early surgical intervention, n (%)	1125 (4.5)	1005 (4.9)	120 (2.8)	<0.0001
Any painful/surgical condition in first 2 days of mechanical ventilation, n (%)	3007 (12.1)	2786 (13.5)	221 (5.2)	
NMR-2000 score, categorised as risk of in-hospital mortality, n (%)^a^				
Low risk	717 (3.2)	606 (3.2)	111 (3.2)	<0.0001
Medium risk	16,785 (75.1)	13,891 (73.7)	2894 (82.5)	
High risk	4842 (21.7)	4341 (23.0)	501 (14.3)	
Level of care of first admitting unit, n (%)				
Neonatal Intensive Care Unit	16,021 (64.6)	12,740 (62.0)	3281 (77.1)	<0.0001
Local Neonatal Unit	7265 (29.3)	6448 (31.4)	817 (19.2)	
Special Care Baby Unit	1527 (6.2)	1372 (6.7)	155 (3.6)	
Length of hospital stay in days, median (IQR)	81 (52–108)	84 (52–111)	73 (49–94)	<0.0001
Death before discharge, n (%)	3836 (15.5)	3395 (16.5)	441 (10.4)	<0.0001

a Missing data: Birth weight z-score, 4 (<0.01%); Caesarean delivery, 879 (3.5%); antenatal steroids, 131 (0.5%); surfactant given, 850 (3.4%); NMR-2000 score, 2471 (10.0%).

Preterm brain injury was identified in 7698/24,815 (31.0%) infants who had MV for >2 consecutive days including 6699/20,561 (32.6%) of those who received an opioid during MV and 999/4254 (23.5%) of those who did not.

Through propensity score matching we identified a matched cohort of 7216 infants (3608 pairs) which was well-balanced across the variables used to calculate the propensity score. Full details of the quality of the match are given in the Supplementary material.

**Fig. 2:**
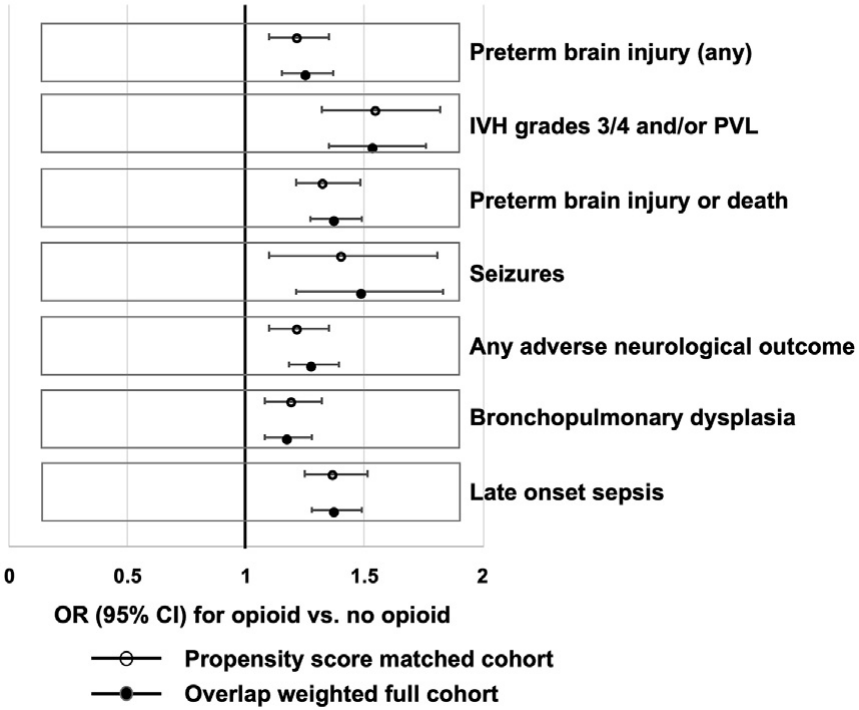
Clinical outcomes in mechanically ventilated infants born at <32 weeks' gestational age who did and not receive opioids (morphine or fentanyl) during mechanical ventilation in England and Wales (2012–2020): adjusted∗ odds ratio and 95% confidence intervals for the whole cohort (n = 21,899) and in 3194 propensity-matched pairs. IVH, intraventricular haemorrhage; PVL, periventricular leukomalacia ∗adjusted for: year of admission; GA completed weeks; z-score <−2 SD; ODN of first admission; unit level of first admission; early onset sepsis; NMR-2000 category; admission temperature; maternal ethnic group; 5 min Apgar score, grouped; multiple birth; any antenatal steroid; received significant resuscitation; received surfactant on day 1; mode of delivery; maternal IMD quintile; maternal age; acute postnatal transfer on day 1/2; total number of days ventilated; need for inotropes during mechanical ventilation.

In the matched cohort, the unadjusted odds of preterm brain injury were higher in those who received opioids compared with those who did not (received opioids, 990/3608 (27.4%) vs. did not receive opioids, 855/3608 (23.7%); OR = 1.22, 95% CI 1.10–1.35). In the matched cohort, the point estimate for more severe grades of IVH (grades 3 or 4) or PVL, and the composite outcome of brain injury or death, were also higher in those who received opioids. The sensitivity analysis of the full cohort using overlap weights showed similar results to the PSM analysis (Fig. 2 and Supplementary Table S9).

### Length of mechanical ventilation

Infants who received opioids had longer duration of MV as compared to those who did not: median (IQR) length of MV for those who received opioids whilst ventilated was 12 (6–27) days and for those who did not receive opioids was 6 (4–11) days (p-value for difference, <0.001). In the matched cohort, this was 8 (5–18) days among those who received opioids and 6 (4–11) days in those who did not (p-value for difference, <0.001).

### Outcomes by duration of opioid exposure

**Fig. 3:**
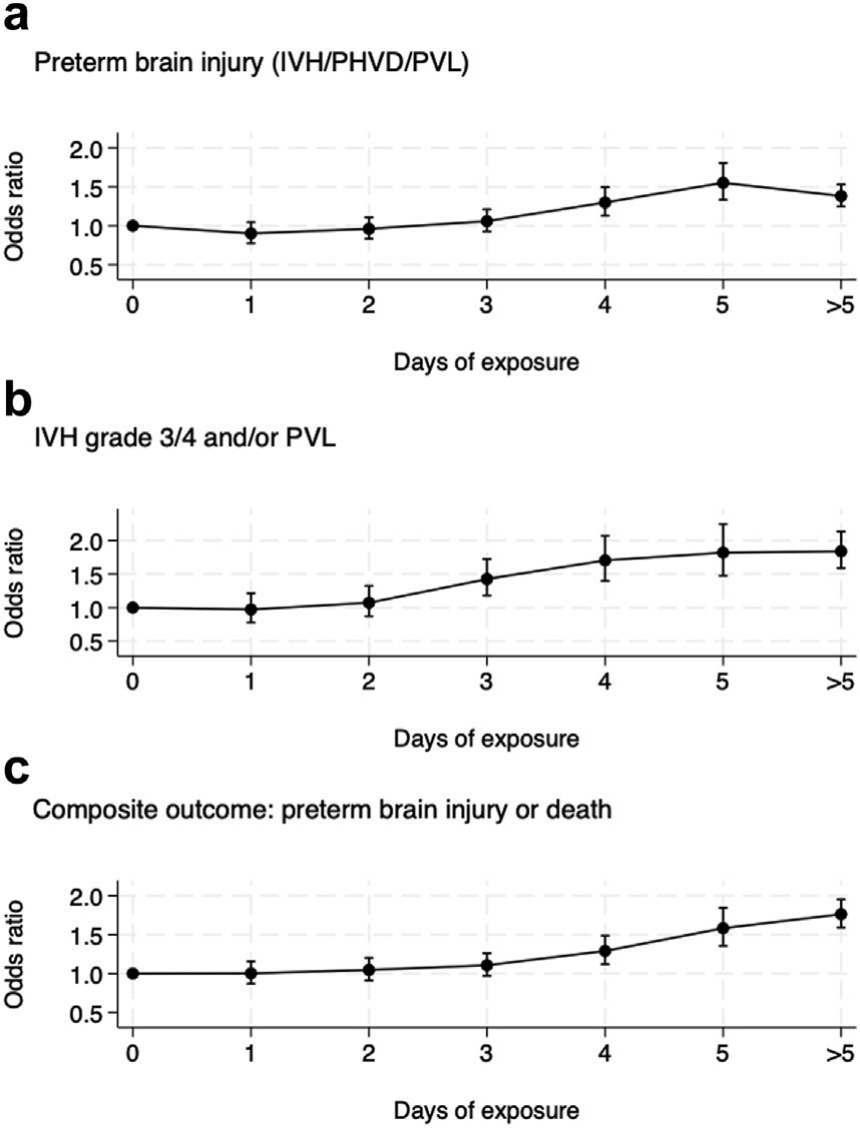
Clinical outcomes in mechanically ventilated infants born at <32 weeks' gestational age by the number of days of exposure to opioids (morphine or fentanyl) during mechanical ventilation in England and Wales (2012–2020): adjusted∗ odds ratio and 95% confidence intervals for the whole cohort.

The adjusted odds of preterm brain injury, severe grades of brain injury, and the composite outcome of brain injury or death increased significantly with increasing number of days of exposure to opioids during MV (Fig. 3 and Supplementary Table S10). In those who received opioids for 5 days, the adjusted odds (95% CI) of brain injury or death was 1.58 (1.36–1.84) as compared with those who did not receive opioids. The numbers of infants who received more than 5 days of opioids was small for each individual number of days of exposure and the odds of the adverse outcomes in this combined group was lower than in those who had opioids for 5 days but remained significantly higher than that in infants who did not receive any opioids.

### Variations in use by neonatal units

Amongst the Level 3 and larger Level 2 neonatal units, the percentage of care days where infants were ventilated ranged from 4.7% to 22.4% and the percentage of MV days when an infant was given an opioid ranged from 11.7% to 79.6%.

## Discussion

In this retrospective propensity score-matched cohort study, we found that, in infants born at <32 weeks' GA who are mechanically ventilated for >2 consecutive days, the odds of preterm brain injury are increased in those who received opioids (vs. those who did not receive opioids).

For the primary analysis, we used a broad definition of preterm brain injury. In our secondary analyses, the magnitude of increase in odds with exposure to opioids for any adverse neurological outcome (including seizures) was similarly raised and the odds for the most severe brain injury (IVH 3/4 and/or PVL) were even higher. We also found that the odds of brain injury increased with increasing duration of exposure to opioids, such that those who had opioid infusions for 5 days had 50% higher odds of brain injury than did those who did not receive opioids, despite accounting for their condition at birth and illness severity during the MV episode.

Between 2012 and 2020, in England and Wales, there has been absolute decline of 3.4% in the percentage of all infants born at <32 weeks' GA who were ventilated at any time during neonatal care. In the same period, the percentage of infants who received morphine whilst ventilated increased by 4.9% and fentanyl use increased three-fold. Overall, opioids were given to 58% of infants while they were mechanically ventilated. In the EUROPAIN audit 70% of infants born at <33 weeks' GA who had MV received opioids.^4^ The overall percentage of MV days on which infants received opioids (67%) was similar to the EUROPAIN study. Importantly, 17% of infants who had >2 days of MV received no analgesia. We also found a similar wide variation in the use of analgesics and sedatives across the neonatal units who contributed data to our study. This wide variation (from 11.7 to 79.6% of MV days) in use of opioids possibly represents uncertainty among practitioners due to lack of evidence to guide appropriate analgesia in preterm infants. Data from term neonates suggests that currently used doses may be over-dosing^19^ and opioid exposure can be reduced by using other analgesics such as paracetamol.^20^ We need clear guidance that recommends use of opioids for analgesia during painful conditions/procedures such as surgery for NEC,^21^ and separately for sedation and/or analgesia during mechanical ventilation.

It is ethically crucial that appropriate and adequate analgesia is given, as needed, to all infants in neonatal care. Analgesics, including opioids currently used to treat pain, are possibly not effective in providing pain relief.^10^ When measured using standardised pain scores, morphine and placebos have similar analgesic effects in mechanically ventilated preterm infants.^10^ In addition, they are potentially neurotoxic with possible association between duration and dose of use and adverse neurological outcomes.^9^ We found that the risk of harm is associated with increasing duration of exposure to opioids. In the NEOPAIN trial, those who did not receive open-label morphine had lower rates of the composite outcome of IVH, PVL or death and similarly, in the placebo group, additional use of open-label morphine was associated with higher rates of this composite outcome.^11^ Higher exposure to morphine has been associated with higher incidence of IVH and PVL and, more recently, higher cumulative doses of fentanyl have been associated with higher incidence of cerebellar injury.^22^ The longer-term impacts of the use of opioids in preterm infants are less clear and may differ by the indication, dose, and duration of use.

Conversely, untreated, repetitive, or prolonged pain itself has adverse effects on brain development.^23^ Studies using lower doses of morphine show no harm and possibly some benefit on executive function at 8–9 years^24^ while those using higher cumulative doses suggest evidence of harm.^23^ We found that infants who received 1 or 2 days of opioids had no increase in odds of brain injury but those with longer exposure had significantly higher odds of adverse outcomes. Selvanathan et al., also reported that associations between early life pain in infants born at <32 weeks' GA and neurodevelopment outcomes at 18–24 months were affected by duration of morphine exposure,^25^ with worse motor scores in those with longer exposure. Steinbauer et al. found that each 100 mg/kg increase in opioid exposure was associated with increased risk of autism spectrum and withdrawn behaviour features at preschool age.^26^ Similarly, Ranger et al., found that ≤32 week infants who were mechanically ventilated were more likely to have internalised behaviour at school age if they had received morphine.^27^ Increase in cumulative opioid exposure is also associated with behavioural disorders in later childhood which may be moderated by genetic variants in morphine metabolism.^28^

We found that 13.5% of those who received opioids also had a painful/surgical condition in the first 2 days of mechanical ventilation while only 5.2% of those who did not receive opioids had any such conditions recorded. Due to the limited information available in the NNRD, we were unable to differentiate between the use of opioids for analgesia due to pain from such conditions and when the opioids were given for sedation and analgesia to facilitate MV alone. We included the presence of surgical/painful conditions in the propensity score matched analysis to reduce bias due to these confounders.

We also found higher odds of late onset sepsis and bronchopulmonary dysplasia in the group that received opioids when compared with those who did not. This raises the possibility that the associations with adverse outcomes may be because the more unwell infants were more likely to have received opioids and were also more likely to have all adverse outcomes. To mitigate this, we used propensity score techniques to create well balanced groups, matched for infant and maternal characteristics, risk of in-hospital mortality measured at birth, and markers for illness and painful or surgical conditions at the time the infant may or may not have received opioids. We did not include the length of MV in the model because the duration of mechanical ventilation can be prolonged by use of opioids.^29^ However, propensity score analyses cannot balance unmeasured characteristics or unknown confounders. We have used robust methodology, accounted for known confounders, and reported outcomes for a very large number of infants, including all eligible infants in England and Wales over a 9-year period. The results are directly relevant to practice across the UK. They are generalisable and applicable to care provided to preterm infants in similar settings where adequately resourced intensive care can be provided to preterm infants. They may also be applicable in low-resource settings where increasing availability of intensive care is improving the survival and outcomes of very preterm and low birth weight infants. The evidence suggests that opioid analgesia at low doses is not associated with adverse neurodevelopmental outcome^24^^,^^25^ in preterm infants subjected to painful experiences such as MV, but that longer duration and larger doses of opioids are harmful. The lack of evidence for the optimal approach could explain the variation in practice among neonatal clinicians. There is uncertainty about which agents to use and the dose and duration at which benefits outweigh harms to the developing brain. The direct estimate of the effect of opioids on ventilated preterm infants can only be determined by conducting adequately powered, randomised controlled trials with long term follow up of the participants. Newer agents, such as dexmedetomidine, which do not impede respiratory drive could be neuroprotective and may be adjuncts for reducing opioid exposure while providing adequate analgesia.^30^ These need investigations to ensure that pain during neonatal care can be alleviated with minimal adverse impact on the developing brain.

## Contributors

LS participated in the concept and design, developed the methodology, performed the analysis of data, participated in interpretation of data, and wrote part of the original draft. DS: participated in the concept and design, interpretation of data, and revised the manuscript. HB: participated in the concept and design, interpretation of data, and revised the manuscript. SO: designed and conceptualised the study, participated in analysis and interpretation of data, wrote the original draft and reviewed and edited the final manuscript. LS and SO accessed and verified the underlying data. All authors had full access to all the data, approve the final manuscript as submitted, and accept responsibility to submit for publication.

## Data sharing statement

Data used in this study are held by the National Neonatal Research Database and can be requested via the Health Data Research UK digital gateway after approval of a proposal, with a signed data access agreement. A data dictionary defining each field in the dataset is available on request from the corresponding author.

## Declaration of interests

We declare no competing interests.
