# Supplementary material for: Association between opioid use during mechanical ventilation in preterm infants and evidence of brain injury: a propensity score-matched cohort study
Source: eClinicalMedicine. 2023 Oct 28;65:102296. doi: 10.1016/j.eclinm.2023.102296 (PMC10632414; doi:10.1016/j.eclinm.2023.102296)
Supplement: Supplementary material [file mmc1.docx]

**Online only supplementary material**

**Title:** Association between opioid use during mechanical ventilation in preterm infants and evidence of brain injury: a propensity-score matched cohort study

Supplementary Table 1. List of codes used to exclude infants with neonatal abstinence syndrome

| neonatal abstinence syndrome  neonatal drug withdrawal (abstinence)  neonatal drug withdrawal (therapeutic use)  observations for neonatal abstinence syndrome  infant affected by maternal drug addiction  maternal drug use  maternal drug use: amphetamines  maternal drug use: cannabis products  maternal drug use: cocaine  maternal drug use: crack cocaine  maternal drug use: opiates  maternal drug use: sedative / hypnotic  other maternal drug/toxicity effects |
| --- |

Supplementary Table 2. Exclusions due to fatal congenital anomalies

| **Anomaly** | **Number excluded** ^a^ |
| --- | --- |
| Anencephaly and similar malformations | <5 |
| Holoprosencephaly | 17 |
| Bilateral renal agenesis/Potter Syndrome | 8 |
| Trisomy 13 (Patau Syndrome) | 7 |
| Trisomy 18 (Edward Syndrome) | 35 |
| Triploidy/tetraploidy/polyploidy | 6 |
| Thanatophoric Dysplasia | <5 |

^a^ Counts <5 suppressed for statistical disclosure control

Supplementary Table 3. Classification of drugs

| Opioids | | Benzodiazepines | Muscle relaxants |
| --- | --- | --- | --- |
| IV | Oral |  |  |
| - Morphine - IV morphine - Morphine infusion - Morphine sulphate - Fentanyl - Alfentanyl - Remifentanyl | - Diamorphine - Oral morphine - Oramorph - Zomorph | - Midazolam | - Atracurium - Pancuronium - Rocuronium - Vecuronium |

Supplementary Table 4. Outcome definitions and derivation

| **Variable** | **Definition and derivation from NNRD** |
| --- | --- |
| Primary outcomes  (defined according to the Royal College of Paediatrics and Child Health National Neonatal Audit Programme (NNAP)^1^ | |
| Neonatal preterm brain injury (IVH/PHVD/PVL) | Any of the following: Germinal matrix/ intraventricular haemorrhage (of any grade) identified by imaging on or before day 28 after birth; Post haemorrhagic ventricular dilatation identified by imaging at any time during neonatal stay; Cystic periventricular leukomalacia identified by imaging at any time during neonatal stay. Derived from recorded diagnoses at admission (NNRD field: DiagnosisAtAdmission), principal diagnoses at discharge (NNRD field: DiagnosesAtDischarge), daily diagnoses (NNRD field: DiagnosesDaily), and cranial ultrasound records (NNRD fields: RightIVH, LeftIVH, PVL). Dichotomous: Any neonatal preterm brain injury=1; No neonatal preterm brain injury=0. |
| Secondary outcomes  (Pre-specified, and selected as outcomes that are important to infants, parents and clinicians, including, where NNRD data allow, items in the Neonatal Core Outcome Set^2^) | |
| IVH grade 3/4 and/or PVL | Intraventricular haemorrhage (grade 3-4) identified by imaging on or before day 28 after birth or cystic periventricular leukomalacia identified by imaging at any time during neonatal stay. Derived as above. Dichotomous: Any IVH grade 3/4 or PVL=1; No IVH grade 3/4 or PVL=0. |
| Neonatal preterm brain injury or death | Any of the following: Germinal matrix/ intraventricular haemorrhage (of any grade) identified by imaging on or before day 28 after birth; Post haemorrhagic ventricular dilatation identified by imaging at any time during neonatal stay; Cystic periventricular leukomalacia identified by imaging at any time during neonatal stay. Derived from recorded diagnoses at admission (NNRD field: DiagnosisAtAdmission), principal diagnoses at discharge (NNRD field: DiagnosesAtDischarge), daily diagnoses (NNRD field: DiagnosesDaily), and cranial ultrasound records (NNRD fields: RightIVH, LeftIVH, PVL).  Or: Final discharge destination recorded as died (NNRD field: DischargeDestination). Dichotomous: Died=1; Survived to discharge=0.  Dichotomous: Any neonatal preterm brain injury or death=1; No neonatal preterm brain injury or death=0. |
| Convulsions | Any recorded seizures or diagnosis of a seizure disorder (excluding benign familial neonatal seizures) during neonatal stay. Derived from recorded diagnoses at admission (NNRD field: DiagnosisAtAdmission), principal diagnoses at discharge (NNRD field: DiagnosesAtDischarge), daily diagnoses (NNRD field: DiagnosesDaily), and daily convulsion indicator (NNRD field: Convulsions). Dichotomous: Any convulsions=1; No convulsions=0. |
| Any adverse neurological outcome | Composite variable representing any neonatal preterm brain injury (IVH any grade/PHVD/PVL) and/or convulsions, derived as above. Dichotomous: Any adverse neurological outcome=1; No adverse neurological outcome=0. |
| Bronchopulmonary dysplasia (BPD) | Receiving respiratory support (any mode of treatment with oxygen) at 36 weeks postmenstrual age. Derived from recorded diagnoses at admission (NNRD field: DiagnosisAtAdmission), principal diagnoses at discharge (NNRD field: DiagnosesAtDischarge), daily diagnoses (NNRD field: DiagnosesDaily), and daily records of respiratory support (NNRD fields: RespiratorySupport, AddedO2, VentilationMode, NonInvasiveRespiratorySupport). Excludes infants who died prior to 36 weeks PMA. Dichotomous: BPD=1; No BPD=0 |
| Late onset sepsis | Pragmatic definition of late onset sepsis:  ≥5 continuous days receiving any of the following antibiotics^3^ (including in combination and changing during the 5 days): Amikacin; Amoxicillin; Amoxicillin; Ampicillin; Augmentin; Azlocillin; Benzyl Penicillin; Cefalexin; Cefotaxime; Ceftazidime; Ceftriaxone; Cefuroxime; Ciprofloxacin; Co-amoxiclav; Flucloxacillin; Flucloxicillin; Gentamicin; Imepenem; Imipenem; Linezolid; Meropenem; Metronidazole; Netilmicin; Oflacillin; Piperacillin; Tazocin; Vancomycin. (NNRD field: DailyDrugs)  Gaps of 1 day in recording of antibiotic use imputed as assumed use if antibiotic use recorded for one or more days both before and after. Recurring courses of antibiotic treatment <3 days apart, or which occurred within the first 3 days of life, were excluded. Dichotomous: LOS=1; No LOS=0. |

Supplementary Table 5. Painful procedures, conditions requiring urgent surgery and major congenital anomalies requiring early surgical intervention

| **Painful procedures** | **Number** ^a^ |
| --- | --- |
| Chest drain in situ | 1,327 |
| Tracheostomy, in situ for ≤7 days | 20 |
|  |  |
| **Painful conditions requiring urgent surgery** | **Number** ^a^ |
| Acute appendicitis | <5 |
| Intestinal obstruction | 21 |
| Intestinal perforation | 99 |
| Necrotising enterocolitis | 1,484 |
| Other perforations | <5 |
| Pneumothorax | 1,006 |
| Surgical treatment for PDA | 31 |
|  |  |
| **Major congenital anomalies requiring early surgical intervention (grouped by ICD-10 code)** | **Number** ^a^ |
| Q01 Encephalocele and similar malformations | 10 |
| Q05 Spina bifida and similar malformations | 29 |
| Q20 Congenital malformations of cardiac chambers and connections | 161 |
| Q21.2 Atrioventricular septal defect | 89 |
| Q21.3 Tetralogy of Fallot | 84 |
| Q22 Congenital malformations of pulmonary and tricuspid valves | 291 |
| Q23 Congenital malformations of aortic and mitral valves | 97 |
| Q25.1 Coarctation of aorta | 120 |
| Q25.2 Atresia of aorta | 38 |
| Q25.3 Stenosis of aorta (AS) | 6 |
| Q25.4 Other congenital malformations of aorta | 37 |
| Q25.5 Atresia of pulmonary artery | <5 |
| Q25.6 Stenosis of pulmonary artery (PS) | 272 |
| Q25.8 Other congenital malformations of great arteries | <5 |
| Q26.2 Total anomalous pulmonary venous connection | 15 |
| Q30.0 Choanal atresia | 31 |
| Q32 Congenital malformations of trachea and bronchus | 120 |
| Q39 Oesophageal atresia | 199 |
| Q41 Congenital absence, atresia and stenosis of small intestine | 159 |
| Q42 Congenital absence, atresia and stenosis of large intestine | 76 |
| Q43.6 Congenital fistula of rectum and anus | 13 |
| Q64.1 Exstrophy of urinary bladder | <5 |
| Q64.2 Posterior urethral valves (PUV) | 27 |
| Q79.0 Congenital diaphragmatic hernia | 73 |
| Q79.1 Eventration of diaphragmatic hernia | 20 |
| Q79.2 Exomphalos | 101 |
| Q79.3 Gastroschisis | 29 |

^a^ Counts <5 suppressed for statistical disclosure control

**Statistical methods: propensity score matching**

*Calculation of propensity scores*

We used the step-wise approach proposed by Imbens and Rubin^4^ to fit a logistic regression model to estimate propensity scores for infants who were ventilated for >2 consecutive days receiving an opioid (morphine and/or fentanyl) for 1 or more days whilst ventilated compared to not receiving an opioid whilst ventilated.

We first compiled a list of all potential background variables based on published literature, clinical experience, and understanding of the data items available within the NNRD. These variables are listed in Supplementary Table 6 below. These background variables were then used to form matched groups for analysis, as described below.

Supplementary Table 6. Definitions and derivation of background variables for propensity score matching

| **Variable** | **Definition and derivation from NNRD** |
| --- | --- |
| Principal background variables requiring exact match | |
| Gestational age (completed weeks) | Gestational age in single completed weeks (NNRD field: GestationWeeks) (range 22-31). |
| Year of admission | Calendar year of first admission to neonatal care (NNRD field: AdmitTimeMonth, AdmitTimeYear), grouped: 2012-2014; 2015-2017; 2018-2020. |
| Highly important background variables | |
| Gestational age (weeks + days) | Continuous variable combining weeks (NNRD field: GestationWeeks) plus days (NNRD field: GestationDays) for propensity score model building. |
| Sex | Phenotypic sex of infant (NNRD field: SexPhenotype). Dichotomous: Male=1, Female=0. |
| Birthweight | Birthweight at time of delivery (NNRD field: Birthweight). Continuous variable measured in grams. |
| Neonatal Operational Delivery Network of first admission | Neonatal Operational Delivery Network derived from NHS provider code of neonatal unit of first admission (NNRD field: ProviderNHSCode). Categorical: East Midlands; East of England; North Central & North East London; North West London; North West; Northern; South East Coast; South London; South West; Thames Valley & Wessex; Wales; West Midlands; Yorkshire & Humber. |
| Moderately important background variables | |
| Small for gestational age | Birthweight-for-age z-score <-2SD. Derived from birthweight (NNRD field: Birthweight), gestational age in single completed weeks (NNRD field: GestationWeeks) and sex (NNRD field: SexPhenotype). Calculated according to UK WHO Preterm Growth References, using the zanthro command in Stata^5^. Dichotomous: <-2SD=1; >=-2SD=0. |
| Level of neonatal care of unit of first admission | Highest level of care offered by neonatal unit of first admission, derived from NHS provider code (NNRD field: ProviderNHSCode). Categorical: Special Care baby unit (SCBU)=Level 1; Local neonatal unit (LNU)=Level 2; Neonatal intensive care unit (NICU)=Level 3. |
| Acute post-natal transfer on day 1/2 | Transferred between neonatal units for continuing care on either the day of birth or the day afterwards. Identified by 2+ daily care records on the same calendar day attributed to different providers (NNRD field: DayDateAnon, DayProviderNHSCode). Dichotomous: Acute post-natal transfer=1; No acute post-natal transfer=0. |
| Received antenatal steroids | Were any antenatal steroids (either complete or incomplete course) given during pregnancy, derived from episodic data (NNRD field: SteroidsAntenatalGiven). Dichotomous: Any antenatal steroids given=1; No antenatal steroids given=0. |
| Multiple birth | Derived from number of fetuses noted during pregnancy (NNRD field: FetusNumber). Categorical: Multiple birth=1; Singleton=0; Missing. |
| Mode of delivery | Infant’s mode of delivery, derived from episodic data (NNRD field: ModeOfDelivery). Categorical: Vaginal=0; Caesarean section=1; Missing. |
| Significant resuscitation | Required significant resuscitation at birth, defined as one or more of cardiac compressions, intubation, adrenaline or other drugs (NNRD field: MethodsOfResuscitation). Dichotomous: Required significant resuscitation=1; No significant resuscitation=0. |
| APGAR score at 5 minutes | APGAR score at 5 minutes of age (NNRD field: apgar_5min), grouped. Categorical: 8-10; 5-7; <5; Missing. |
| Risk of in-hospital mortality | NMR-2000 score^6^ predicting risk of in-hospital mortality, grouped. Derived from record of oxygen saturation at first admission (NNRD field: AdmissionOxygenSaturation), respiratory support on days 1 and 2 (NNRD fields: RespiratorySupport, AddedO2, VentilationMode, NonInvasiveRespiratorySupport) and birthweight (NNRD field: Birthweight). Categorical: High risk; Medium risk; Low risk; Missing. |
| Temperature on admission to neonatal care | Temperature in degrees Celsius on first admission to neonatal care (NNRD field: AdmitTemperature), grouped. Categorical: <36.5; 36.5-37.5; >37.5; Missing. |
| Received surfactant on day 1 | Received pulmonary surfactant on the day of birth, either in the delivery room (NNRD field: SurfactantGivenResuscitation) or on the neonatal unit (NNRD field: DrugsDaily). Categorical: Received surfactant=1; Did not receive surfactant=0; Missing. |
| Received inotropes whilst ventilated | Received an inotrope (dopamine, dobutamine, adrenaline, noradrenaline) on one or more days whilst mechanically ventilated (NNRD fields: InotropesToday, DailyDrugs). Categorical: Received inotropes=1; Did not receive inotropes=0. |
| Surgical/painful congenital anomaly, diagnosis or procedure | Diagnosis of major congenital anomaly requiring early surgical intervention and/or record of a painful procedure or diagnosis requiring urgent surgery in the first two days of MV. Derived from recorded diagnoses at admission (NNRD field: DiagnosisAtAdmission), principal diagnoses at discharge (NNRD field: DiagnosesAtDischarge), daily diagnoses (NNRD field: DiagnosesDaily), principal procedures during stay (NNRD field: PrincipalProceduresDuringStay), chest drain in situ (ChestDrain), tracheostomy in situ (DayTracheostomy), treatment for PDA (TreatmentForPDA). Dichotomous: Any relevant condition=1; No relevant condition=0. |
| Microbiologically confirmed or clinically suspected early onset sepsis | Pragmatic definition of early onset sepsis, including one or more of the following:   1. Pure growth of a clearly pathogenic organism from a blood or CSF culture in the first 72 hours after birth (NNRD fields: CultureDateTimeAnon, Sampletype, Pathogen) 2. Mixed growth, or pure growth of an organism of uncertain significance (including skin commensals), from the blood or CSF *AND* ≥3 clinical signs at the time of sampling in the first 72 hours after birth (NNRD fields: CultureDateTimeAnon, Sampletype, Pathogen, Clinicalsigns) 3. ≥5 continuous days, starting on days 1-3, receiving any of the following antibiotics^3^ (including in combination and changing during the 5 days): Amikacin; Amoxicillin; Amoxicillin; Ampicillin; Augmentin; Azlocillin; Benzyl Penicillin; Cefalexin; Cefotaxime; Ceftazidime; Ceftriaxone; Cefuroxime; Ciprofloxacin; Co-amoxiclav; Flucloxacillin; Flucloxicillin; Gentamicin; Imepenem; Imipenem; Linezolid; Meropenem; Metronidazole; Netilmicin; Oflacillin; Piperacillin; Tazocin; Vancomycin. (NNRD field: DailyDrugs). Gaps of 1 day in recording of antibiotic use imputed as assumed use if antibiotic use recorded for one or more days both before and after   Dichotomous: Microbiologically confirmed or clinically suspected early onset sepsis=1; No microbiologically confirmed or clinically suspected early onset sepsis=0. |
| Total number of days of care | Number of days of neonatal care prior to final discharge. Continuous variable. |
| Maternal age | Age of mother, derived from mother’s calendar year of birth (NNRD field: BirthYearMother), grouped. Categorical: <16; 16-25; 26-35; 36+, Missing. |
| Maternal ethnic group | Mother’s ethnic group (NNRD field: MumEthnicity). Categorical: White; Mixed; Asian/Asian British; Black/Black British; Other; Missing. |
| Index of Multiple Deprivation quintile | Quintile of Index of Multiple Deprivation, deprived from mother’s Lower Level Super Output Area (NNRD field: PostCodeMotherLSOA). Categorical: Most deprived; quintile 2; quintile 3; quintile 4; Least deprived; Missing. |

Two variables were selected as principal background variables, the combination of which was used to define groups within which matching then took place:

1. Gestational age in single completed weeks (22 to 31 weeks)
2. Year of admission, grouped: 2012-2014; 2015-2017; 2018-2020

Four variables were deemed to be highly important background variables which were included in the logistic regression model *a-priori*:

1. Gestational age as a continuous variable measured in weeks plus days
2. Sex
3. Birthweight as a continuous variable measured in grams
4. Neonatal Operational Delivery Network of first admission

The remaining variables were classed as moderately important background variables. Logistic regression models were fitted with each of these variables added individually. The model with the largest value of the chi-squared statistic was adopted, if the test statistic exceeded 1∙0. This cycle was repeated, with all remaining moderately important background variables added individually, until none of the chi-squared test statistics for including a variable exceeded 1∙0 or until all variables had been selected for inclusion in the model. This process led to the identification of 19 main effects for inclusion in the propensity score model (all variables in Supplementary Table 6 with the exception of receipt of antenatal steroids, small-for-gestational-age and maternal age).

Next, interactions between background variables were identified for inclusion in the model. The main effects included in the model were sorted in descending order of the absolute value of their t-ratios (|estimate/st.error|). Starting with the variable with the highest t-ratio we listed all potential interactions which were candidates for inclusion in the model. Continuous variables could be interacted with themselves but binary and categorical variables could not. We added potential interactions individually to the model and selected up to two interactions with the largest value of the chi-squared statistic, if the test statistic exceeded 2∙71. This process was repeated with each of the main effects in turn. This led to identification of 38 interactions for inclusion in the propensity score model.

The logistic regression model including main effects and interactions yielded propensity scores – the estimated probability of an infant receiving an opioid given their background characteristics. Infants with extreme propensities outside of the overlap between intervention and control groups were trimmed from the dataset (i.e., infants who did not receive an opioid who had a propensity score less than the smallest value among those who did, and infants who did receive an opioid who had a propensity score greater than the smallest value among those who did not).

The entire modelling procedure was then repeated again on the trimmed dataset, and the dataset was trimmed for a second time based on the estimated propensity scores. The propensity model identified in this second round of model identification included the same 19 main effects and, with some slight differences to round 1, 38 interactions.

*Matching on propensity scores*

Within each of 30 background groups (10 gestational age weeks multiplied by 3 year of admission periods), infants who received an opioid were matched to infants who did not receive an opioid whilst ventilated. We used nearest-neighbour matching based on the logit of the propensity score, with a caliper width of 0∙2 of the standard deviation of the logit of the propensity score^7^. This yielded a matched cohort of an intervention group (who received an opioid) and a control group (who did not receive an opioid).

Supplementary Figure 1 shows the distribution of propensity scores for infants who received an opioid and infants who did not receive an opioid whilst ventilated, before and after propensity score matching. Logistic regression was used in the matched cohort to estimate odds ratios for adverse outcomes in infants who received an opioid compared to infants who did not.

**Supplementary Figure 1. Propensity score distribution for infants ventilated for >2 consecutive days who received an opioid whilst ventilated and infants ventilated for >2 consecutive days who did not receive an opioid whilst ventilated, before and after propensity score matching**

*Assessment of the quality of the match*

The quality of the match is assessed by the balance of background variables between the groups who did and did not receive an opioid in the matched cohort. We calculated standardised differences between the two groups in rates (for binary and categorical background variables) and means (for continuous variables), with the difference divided by the pooled standard deviation of the variable (pooled across the two groups). Supplementary Table 7 shows these standardised differences, for the cohort before matching and for the matched cohort. Covariates can considered balanced if the standardised differences are between -0∙2 and 0∙2^8,9^; in this regard the matched groups were considered well balanced. Prior to matching the overall standardised mean bias was 11∙3%. This reduced to 1∙6% after matching, less than the 5% cut-off considered acceptable^8,9^.

Supplementary Table 7. Standardised differences in covariates between opioid and no opioid groups, before and after matching

| Characteristic | Standardised difference: full cohort | Standardised difference: matched cohort |
| --- | --- | --- |
| Gestational age, days (Median, IQR) | 0.21 | -0.00 |
| Birthweight, grams (Median, IQR) | 0.15 | -0.01 |
| Number of days of care (Median, IQR) | -0.30 | 0.03 |
| Year of admission: 2012-2014 | 0.19 | 0.00 |
| Year of admission: 2015-2017 | -0.11 | 0.00 |
| Year of admission: 2018-2020 | -0.09 | 0.00 |
| Gestational age: 22 weeks | -0.02 | 0.00 |
| Gestational age: 23 weeks | -0.15 | 0.00 |
| Gestational age: 24 weeks | -0.18 | 0.00 |
| Gestational age: 25 weeks | -0.06 | 0.00 |
| Gestational age: 26 weeks | 0.06 | 0.00 |
| Gestational age: 27 weeks | 0.05 | 0.00 |
| Gestational age: 28 weeks | 0.06 | 0.00 |
| Gestational age: 29 weeks | 0.08 | 0.00 |
| Gestational age: 30 weeks | 0.07 | 0.00 |
| Gestational age: 31 weeks | 0.02 | 0.00 |
| Neonatal ODN: East Midlands | -0.12 | 0.02 |
| Neonatal ODN: East of England | -0.27 | 0.03 |
| Neonatal ODN: North Central and North East London | -0.03 | 0.03 |
| Neonatal ODN: North West London | 0.23 | -0.03 |
| Neonatal ODN: North West | 0.27 | -0.02 |
| Neonatal ODN: Northern | 0.15 | -0.01 |
| Neonatal ODN: South East Coast | -0.13 | 0.01 |
| Neonatal ODN: South London | 0.01 | -0.01 |
| Neonatal ODN: South West | -0.10 | 0.01 |
| Neonatal ODN: Thames Valley and Wessex | -0.06 | -0.00 |
| Neonatal ODN: Wales | -0.01 | -0.01 |
| Neonatal ODN: West Midlands | 0.09 | -0.02 |
| Neonatal ODN: Yorkshire and Humber | -0.23 | 0.03 |
| Level of Care of first admitting unit: Level 1 | -0.14 | 0.00 |
| Level of Care of first admitting unit: Level 2 | -0.28 | 0.06 |
| Level of Care of first admitting unit: Level 3 | 0.33 | -0.05 |
| Sex: Male | 0.05 | -0.00 |
| Multiple birth | 0.07 | -0.03 |
| Received antenatal steroids | 0.04 | -0.02 |
| Mode of delivery: Vaginal | -0.09 | -0.02 |
| Mode of delivery: Caesarian section | 0.07 | 0.02 |
| Mode of delivery: Missing | 0.05 | -0.00 |
| Significant resuscitation at birth | -0.04 | -0.02 |
| Apgar score at 5 minutes: 8-10 | 0.13 | -0.01 |
| Apgar score at 5 minutes: 5-7 | -0.07 | 0.01 |
| Apgar score at 5 minutes: <5 | -0.14 | 0.01 |
| Apgar score at 5 minutes: Missing | 0.03 | -0.01 |
| Acute postnatal transfer on day 1/2 | -0.37 | 0.04 |
| NMR-2000 score, categorised: High risk | -0.25 | 0.05 |
| NMR-2000 score, categorised: Medium risk | 0.01 | -0.01 |
| NMR-2000 score, categorised: Low risk | -0.02 | 0.04 |
| NMR-2000 score, categorised: Missing | 0.28 | -0.05 |
| Temperature at first admission: <36.5 | -0.07 | -0.03 |
| Temperature at first admission: 36.5-37.5 | 0.04 | 0.02 |
| Temperature at first admission: >37.5 | 0.06 | 0.01 |
| Temperature at first admission: Missing | -0.05 | 0.00 |
| Received surfactant on day 1: No | 0.07 | 0.02 |
| Received surfactant on day 1: Yes | -0.19 | -0.02 |
| Received surfactant on day 1: Missing | 0.22 | -0.01 |
| Early onset sepsis | -0.32 | 0.04 |
| Received inotropes whilst ventilated | -0.70 | 0.08 |
| Surgical congenital anomaly and/or painful/surgical condition | -0.30 | 0.05 |
| Maternal ethnic group: White | -0.09 | -0.03 |
| Maternal ethnic group: Mixed | 0.05 | 0.03 |
| Maternal ethnic group: Asian/Asian British | 0.05 | 0.00 |
| Maternal ethnic group: Black/Black British | 0.03 | -0.01 |
| Maternal ethnic group: Other | 0.04 | 0.01 |
| Maternal ethnic group: Missing | 0.01 | 0.03 |
| Index of Multiple Deprivation quintile: Most deprived | 0.08 | -0.00 |
| Index of Multiple Deprivation quintile: 2 | -0.02 | 0.01 |
| Index of Multiple Deprivation quintile: 3 | -0.08 | 0.03 |
| Index of Multiple Deprivation quintile: 4 | -0.04 | 0.00 |
| Index of Multiple Deprivation quintile: Least deprived | -0.04 | 0.00 |
| Index of Multiple Deprivation quintile: Missing | 0.06 | -0.04 |
| Maternal age: <26 | -0.05 | -0.03 |
| Maternal age: 26-35 | 0.01 | 0.00 |
| Maternal age: 36+ | 0.03 | 0.02 |
| Maternal age: Missing | 0.01 | 0.01 |

Supplementary Figure 2. Infants included in the study, by gestational age subgroup

Supplementary Figure 3. Percentage of infants admitted to neonatal units in England and Wales (2012-2020) who were mechanically ventilated

Supplementary Table 8.Use of opioids, benzodiazepines, and muscle relaxants in neonatal care of infants born at <32 weeks' GA in England and Wales (2012-2020)

|  | All infants | <28 weeks’ GA | 28-31 weeks’ GA |
| --- | --- | --- | --- |
| Number (%) infants who received the drug | | | |
| Any opioid | 26,900 (40.0) | 14,675 (69.7) | 12,225 (26.5) |
| Any morphine | 25,780 (38.4) | 14,418 (68.5) | 11,362 (24.6) |
| IV morphine | 25,594 (38.1) | 14,294 (67.9) | 11,300 (24.5) |
| Oral morphine | 3,199 (4.8) | 2,761 (13.1) | 438 (0.9) |
| Fentanyl | 5,050 (7.5) | 2,736 (13.0) | 2,314 (5.0) |
| Midazolam | 2,226 (3.3) | 1,662 (7.9) | 564 (1.2) |
| Opioid + midazolam (same day) | 2,041 (3.0) | 1,525 (7.2) | 516 (1.1) |
| Any muscle relaxant | 9,123 (13.6) | 5,992 (28.5) | 3,131 (6.8) |
| Atracurium | 4,084 (6.1) | 2,544 (12.1) | 1,540 (3.3) |
| Pancuronium | 3,758 (5.6) | 2,632 (12.5) | 1,126 (2.4) |
| Rocuronium | 1,041 (1.5) | 747 (3.5) | 294 (0.6) |
| Vecuronium | 2,417 (3.6) | 1,685 (8.0) | 732 (1.6) |
| Total number of days of use of the drug, median (IQR) | | | |
| Any opioid | 4 (2-12) | 8 (3-21) | 2 (1-5) |
| Any morphine | 5 (2-13) | 8 (3-21) | 3 (1-5) |
| IV morphine | 4 (2-12) | 8 (3-18) | 3 (1-5) |
| Oral morphine | 12 (5-21) | 12 (5-22) | 8 (3-17) |
| Fentanyl | 1 (1-2) | 1 (1-2) | 1 (1-1) |
| Midazolam | 3 (1-7) | 4 (2-8) | 3 (1-6) |
| Opioid + midazolam (same day) | 3 (2-7) | 4 (2-8) | 3 (1-6) |
| Any muscle relaxant | 2 (1-4) | 2 (1-5) | 1 (1-3) |
| Atracurium | 2 (1-4) | 2 (1-5) | 1 (1-3) |
| Pancuronium | 1 (1-2) | 2 (1-3) | 1 (1-2) |
| Rocuronium | 2 (1-4) | 2 (1-4) | 2 (1-4) |
| Vecuronium | 2 (1-4) | 2 (1-4) | 2 (1-3) |
| GA, gestational age; IQR, interquartile range | | | |

Supplementary Table 9. Outcomes in infants born at <32 weeks’ GA who were mechanically ventilated for >2 consecutive days who did and did not receive an opioid for 1+ days

| **Outcome** | **All infants ventilated for >2**  **consecutive days** | | **Propensity score matched cohort** | | | **Full cohort with overlap weighting** |
| --- | --- | --- | --- | --- | --- | --- |
|  | **Received an opiod for 1+ days** | **Did not receive an opiod** | **Received an opiod for 1+ days** | **Did not receive an opiod** | **Odds Ratio**  **(95% CI)** | **Odds Ratio**  **(95% CI)** |
| Number of infants, n | 20,561 | 4,254 | 3,608 | 3,608 | 7,216 | 24,222^a^ |
| Preterm brain injury (any IVH/PHVD/PVL), n (%) | 6,699 (32.6) | 999 (23.5) | 990 (27.4) | 855 (23.7) | 1.22 (1.10 to 1.35)^b^ | 1∙26 (1∙15 to 1∙37)^b^ |
| IVH grade 3/4 and/or PVL, n (%) | 3,053 (14.8) | 324 (7.6) | 422 (11.7) | 284 (7.9) | 1∙55 (1∙32 to 1∙82)^b^ | 1∙54 (1∙35 to 1∙76)^b^ |
| Preterm brain injury or death | 8,424 (41.0) | 1,233 (29.0) | 1,245 (34.5) | 1,021 (28.3) | 1∙33 (1∙21 to 1∙48)^b^ | 1∙38 (1∙27 to 1∙49)^b^ |
| Convulsions, n (%) | 1,297 (6.3) | 133 (3.1) | 156 (4.3) | 112 (3.1) | 1∙41 (1∙10 to 1.81)^b^ | 1∙49 (1∙21 to 1∙83)^b^ |
| Any adverse neurological outcome, n (%) | 7,288 (35.4) | 1,072 (25.2) | 1,060 (29.4) | 918 (25.4) | 1∙22 (1∙10 to 1∙35)^b^ | 1∙28 (1∙18 to 1∙39)^b^ |
| BPD, n (%) | 12,725 (72.5) | 2,315 (60.6) | 2,030 (64.5) | 1,975 (60.3) | 1∙20 (1∙08 to 1∙32)^b^ | 1∙18 (1∙08 to 1∙28)^b^ |
| Late onset sepsis, n (%) | 12,254 (59.6) | 1,826 (42.9) | 1,933 (53.6) | 1,647 (45.6) | 1∙37 (1∙25 to 1∙51)^b^ | 1∙38 (1∙28 to 1∙49)^b^ |

^a^593 infants were not included in this analysis as their propensity score could not be calculated due to small numbers of observations for some combinations of background variables

^b^p<0∙05 after Bonferroni adjustment

BPD, Bronchopulmonary dysplasia; IVH, intraventricular haemorrhage; PHVD, post haemorrhagic ventricular dilatation; PVL, periventricular leukomalacia∙

Supplementary Table 10∙ Adjusted odds ratio for adverse outcomes in infants born at <32 weeks’ GA who were mechanically ventilated for >2 consecutive days by number of days of opioids received

| Days of opioid exposure | Number of infants | Preterm brain injury  (any IVH/PHVD/PVL) | | IVH grade 3/4 and/or PVL | | Composite outcome: brain injury or death | |
| --- | --- | --- | --- | --- | --- | --- | --- |
|  |  | n (%) | aOR^a^ (95% CI) | n (%) | aOR^a^ (95% CI) | n (%) | aOR^a^ (95% CI) |
| 0 | 4,254 | 999 (23.5) | Ref | 324 (7.6) | Ref | 1,233 (29.0) | Ref |
| 1 | 1,881 | 443 (23.6) | 0.90 (0.78 to 1.05) | 172 (9.1) | 0.97 (0.78 to 1.21) | 585 (31.1) | 1.00 (0.87 to 1.16) |
| 2 | 2,016 | 486 (24.1) | 0.96 (0.83 to 1.11) | 190 (9.4) | 1.07 (0.87 to 1.32) | 644 (31.9) | 1.05 (0.91 to 1.20) |
| 3 | 2,374 | 623 (26.2) | 1.06 (0.93 to 1.21) | 296 (12.5) | 1.43 (1.18 to 1.72) | 804 (33.9) | 1.11 (0.97 to 1.26) |
| 4 | 1,882 | 580 (30.8) | 1.30 (1.13 to 1.50) | 263 (14.0) | 1.70 (1.40 to 2.07) | 704 (37.4) | 1.29 (1.12 to 1.49) |
| 5 | 1,425 | 498 (34.9) | 1.55 (1.33 to 1.81) | 217 (15.2) | 1.82 (1.47 to 2.25) | 588 (41.3) | 1.58 (1.36 to 1.84) |
| >5 | 10,983 | 4,069 (37.0) | 1.38 (1.25 to 1.53) | 1,915 (17.4) | 1.84 (1.59 to 2.13) | 5,099 (46.4) | 1.76 (1.59 to 1.95) |

^a^Adjusted for: sex; gestational age, completed weeks; birthweight-for-age z-score <-2SD; NMR 2000 risk category; 5 minute Apgar score categorised; unit level of first admission; year of birth; number of days of care; inotropes received whilst ventilated; congenital anomaly requiring early surgical intervention and/or painful/surgical condition on first 2 days of mechanical ventilation

**References for online supplementary material**

1 Royal College of Paediatrics and Child Health. National Neonatal Audit Programme (NNAP) Measures. https://www.rcpch.ac.uk/work-we-do/clinical-audits/nnap/measures (accessed June 6, 2023).

2 Webbe JWH, Duffy JMN, Afonso E, *et al.* Core outcomes in neonatology: development of a core outcome set for neonatal research. *Arch Dis Child - Fetal Neonatal Ed* 2020; **105**: 425–31.

3 Webbe JWH, Longford N, Battersby C, *et al.* Outcomes in relation to early parenteral nutrition use in preterm neonates born between 30 and 33 weeks’ gestation: a propensity score matched observational study. *Arch Dis Child Fetal Neonatal Ed* 2022; **107**: 131–6.

4 Imbens G, Rubin DB. Causal Inference for Statistics, Social, and Biomedical Sciences: An Introduction. New York: Cambridge University Press, 2015.

5 Vidmar S, Carlin J, Hesketh K. Standardizing anthropometric measures in children and adolescents with new functions for egen. *Stata J* 2004; **4**: 50–5.

6 Medvedev MM, Brotherton H, Gai A, *et al.* Development and validation of a simplified score to predict neonatal mortality risk among neonates weighing 2000 g or less (NMR-2000): an analysis using data from the UK and The Gambia. *Lancet Child Adolesc Health* 2020; **4**: 299–311.

7 Austin PC. Optimal caliper widths for propensity‐score matching when estimating differences in means and differences in proportions in observational studies. *Pharm Stat* 2011; **10**: 150–61.

8 Rosenbaum PR, Rubin DB. Constructing a Control Group Using Multivariate Matched Sampling Methods That Incorporate the Propensity Score. *Am Stat* 1985; **39**: 33.

9 Caliendo M, Kopeinig S. Some practical guidance for the implementation of propensity score matching. *J Econ Surv* 2008; **22**: 31–72.
