# Supplementary material for: Association between opioid use during mechanical ventilation in preterm infants and evidence of brain injury: a propensity score-matched cohort study
Source: eClinicalMedicine. 2023 Oct 28;65:102296. doi: 10.1016/j.eclinm.2023.102296 (PMC10632414; doi:10.1016/j.eclinm.2023.102296)
Supplement: Protocol.docx [file mmc2.docx]

| **PROTOCOL** | |
| --- | --- |
| Study name | Association between opioid use during mechanical ventilation in preterm infants and evidence of brain injury: a propensity-score matched cohort study |
| Funder | None |
| Chief investigator | Dr Shalini Ojha |
| Statistician | Dr Lisa Szatkowski |

| **BACKGROUND** | |
| --- | --- |
| Objectives | - Describe patterns of use of opioids during mechanical ventilation in infants born at <32 weeks of gestation - Investigate the association between use of opioids, duration of exposure, and evidence of preterm brain injury. |

| **DATA DETAILS** | |
| --- | --- |
| Study type | Retrospective cohort study with propensity score matching |
| Data source | Data source: the National Neonatal Research Database (NNRD)   - a repository of clinical data from all admissions to NHS neonatal units in England, Wales and Scotland since 2012 - over 400 data items entered at the point of care - data entered by clinical staff into electronic patient records and extracted by the Neonatal Data Analysis Unit (NDAU) at Imperial College, London - data anonymised, cleaned, and combined - approval from NHS Research Ethics Committee (10/80803/151) and the Caldicott Guardians of NHS Trusts for research use |
| Study population | Extremely and very preterm infants admitted to neonatal units in England and Wales who contribute data to the NNRD |
| Inclusion/exclusion criteria | Inclusion criteria:   - <32 completed weeks’ GA at birth - admitted between 01 January 2012 and 31 December 2020 - mechanically ventilated for >2 consecutive days   Exclusion criteria:   - missing data on GA, sex, birthweight or final discharge destination - missing data for one or more episodes of care during neonatal stay - implausible birthweight for GA z-score value <-4SD or >+4SD - first admission to neonatal care >24 hours after birth - record of neonatal abstinence symptoms or syndrome and/or maternal opioid use |
| Exposure variables | Mechanically ventilated on one or more days during neonatal stay  Prescribed one or more of the following on one or more days during neonatal stay:   - opioids: IV morphine (morphine, morphine infusion, morphine sulphate); oral morphine (diamorphine, Oramorph, Zomorph); fentanyl (fentanyl, alfentanyl, remifentanyl) - sedatives: midazolam - muscle relaxants: atracurium, pancurnium, rocuronium, vercuronium |
| Primary outcome measure | Preterm brain injury, defined the National Neonatal Audit Programme definition as intraventricular haemorrhage (IVH) of any grade identified by imaging on or before day 28, cystic periventricular leukomalacia (PVL) or post haemorrhagic ventricular dilatation (PHVD) identified by any imaging during neonatal stay[1]. |
| Secondary outcome measures | Severe brain injury, defined as IVH grade 3-4 identified by imaging on or before day 28 after birth or cystic periventricular leukomalacia identified by imaging at any time during neonatal stay.  Composite outcome of any brain injury or death.  Other relevant outcomes from Neonatal Core Outcome Set[2]:   - convulsions - any adverse neurological outcomes - bronchopulmonary dysplasia - late onset sepsis |
| Covariates | Data on the following covariates will be extracted to describe the study population and allow for adjustment for confounders:   - Gestational age at birth (GA) in completed weeks, and grouped according to convention (extremely preterm – 22-27 weeks, very preterm – 28-31 weeks) - Sex (male, female) - Birthweight (g) - Birthweight for gestational age z-score, defined according to the UK-WHO Preterm Growth Reference - Multiple birth (singleton, multiple) - Discharge destination (home, ward, transferred for ongoing specialist care, died) - Length of hospital stay (days) - Mode of delivery (vaginal, caesarean) - Required significant resuscitation at birth (cardiac compressions/ intubation/ adrenaline/ other drugs) - Apgar score at 5 minutes - Acute post-natal inter-hospital transfer on day 1 or 2 of life (yes, no) - NMR-2000[3] score indicating risk of in-hospital mortality (low risk, medium risk, high risk) - Major surgical or congenital anomaly (yes, no) - Required mechanical ventilation on day 1 of life (yes, no) - Received surfactant on day 1 of life (yes, no) - Mother received any antenatal steroids (yes, no) - Maternal age (years) - Maternal ethnic group (White, Mixed, Asian/Asian British, Black/Black British, Other) - Maternal Index of Multiple deprivation (quintile) - Operational Delivery Network of first admitting unit - Level of care of first admitting unit (Level 3 – NICU, Level 2 – LNU, Level 3 – SCBU) |
| Approach to dealing with missing data | Based on previous studies using NNRD data, the amount of missing data is generally expected to be small. Infants with missing data on the key characteristics of gestational age, sex, birthweight and discharge destination will be excluded, as missing data for these outcomes calls into question the quality of data recording for the infant’s entire electronic patient record.  We will not impute data on the diagnosis of adverse outcomes – where there is no evidence of any given adverse outcome recorded in the medical record this will be taken as the outcome not having occurred. Missing data will be treated as a separate category for variables in the propensity score matching model. |

| **ANALYTICAL STRATEGY** |
| --- |
| Data preparation and description of study population  After data cleaning and derivation of the variables described above, the number of infants with missing data across key variables will be tabulated and the total number of exclusions and final study population size determined.  Description of drug use  Overall, and by gestational age sub-group (extremely preterm, <28 weeks’ GA; very preterm, 28-31 weeks’ GA) and by year of birth, we will count the number and percentage of infants who received any mechanical ventilation during neonatal care and the count and percentage of infants who received >2 consecutive days of mechanical ventilation. We will describe the number of days of mechanical ventilation per infant overall, using median plus interquartile range (IQR) or mean plus standard deviation (SD), as appropriate to the distribution of the data.  We will count the number and percentage of infants who received morphine and/or fentanyl at least once at any point during their neonatal stay and at any point whilst mechanically ventilated. We will quantify the cumulative days of use of morphine and/or fentanyl and the percentage of days on which infants were mechanically ventilated where these drugs were prescribed. Use of sedatives and muscle relaxants will be similarly described.  We will calculate the total number of mechanical ventilation days individually by unit, for all Level 3 units (Neonatal Intensive Care Units) and larger Level 2 units (Local Neonatal Units, defined as units who delivered at least 15,000 patient care days per year – approximately the median workload across all Level 2 units). By unit, we will calculate the percentage of mechanical ventilation days where infants received an opioid.  Association between opioid use during mechanical ventilation and the primary and secondary outcomes  We will compare demographic and clinical characteristics of infants who were mechanically ventilated for >2 consecutive days who did, and did not, receive an opioid during mechanical ventilation. Overall, and by opioid exposure group, we will count the number and percentage of infants who had each of the primary and pre-specified secondary outcomes.  Using the methods outlined by Imbens and Rubin[4] we will use propensity score matching to identify pairs of infants with similar demographic and clinical characteristics, matching one infant who received an opioid during mechanical ventilation with an otherwise similar infant who did not. Infants will be matched exactly on gestational age in completed weeks and year of admission. The following variables will be considered to be highly important background variables, included in the propensity score model a-priori: gestational age (continuous variable in weeks + days); sex; birthweight in grams; Neonatal Operational Delivery Network of first admission. The following variables will be considered moderately important background variables and will be assessed for inclusion in the propensity score model: mother received antenatal steroids; mode of delivery; significant resuscitation; Apgar score at 5 minutes; multiple birth; birthweight-for-age z-score <-2SD; NMR-2000 category; acute post-natal transfer on day ½; temperature on admission to neonatal care; received surfactant on day 1; received inotropes whilst ventilated; microbiologically confirmed or clinically suspected early onset sepsis; total number of days of care; maternal age; maternal ethnic group; Index of Multiple Deprivation quintile; unit level of first admission.  In propensity score matched cohort we will use logistic regression to calculate odds ratios for the association between receiving opioids during mechanical ventilation and the primary and secondary outcomes. As a sensitivity analysis, we will use overlap weights[5] derived from the propensity scores to weight the calculation of odds ratios for adverse outcomes in the full cohort, such that all infants contribute to the analysis with their contribution proportional to their overlap weight.  We will use conventional multivariable logistic regression to describe the association between the number of days of opioid exposure during mechanical ventilation and the odds of adverse outcomes. Infants not exposed to opioids will be used as the reference group. If numbers allow, we will analyse opioid use by increasing number of individual days of exposure. |

| **REFERENCES** |
| --- |
| 1 RCPCH. National Neonatal Audit Programme (NNAP) Summary report on 2021 data. 2022. https://www.rcpch.ac.uk/sites/default/files/2022-10/nnap_summary_report_on_2021_data.pdf  2 Webbe JWH, Duffy JMN, Afonso E, *et al.* Core outcomes in neonatology: development of a core outcome set for neonatal research. *Arch Dis Child - Fetal Neonatal Ed* 2020;**105**:425–31. doi:10.1136/archdischild-2019-317501  3 Medvedev MM, Brotherton H, Gai A, *et al.* Development and validation of a simplified score to predict neonatal mortality risk among neonates weighing 2000 g or less (NMR-2000): an analysis using data from the UK and The Gambia. *Lancet Child Adolesc Health* 2020;**4**:299–311. doi:10.1016/S2352-4642(20)30021-3  4 Imbens G, Rubin DB. *Causal Inference for Statistics, Social, and Biomedical Sciences: An Introduction*. New York: : Cambridge University Press 2015.  5 Li F, Thomas LE. Addressing Extreme Propensity Scores via the Overlap Weights. *Am J Epidemiol* Published Online First: 5 September 2018. doi:10.1093/aje/kwy201 |
